# Physicochemical characterisation of barley straw treated with sodium hydroxide or urea and its digestibility and in vitro fermentability in ruminants

**DOI:** 10.1038/s41598-022-24738-w

**Published:** 2022-11-29

**Authors:** Martin Bachmann, Siriwan D. Martens, Yann Le Brech, Gwendal Kervern, Robin Bayreuther, Olaf Steinhöfel, Annette Zeyner

**Affiliations:** 1 Institute of Agricultural and Nutritional Sciences, Martin Luther University Halle-Wittenberg, Halle (Saale), Germany; 2 Saxon State Office for Environment, Agriculture and Geology, Köllitsch, Germany; 3 CNRS, ENSIC-LRGP, Université de Lorraine, Nancy, France; 4 CNRS, CRM2, Université de Lorraine, Nancy, France

**Keywords:** Biochemistry, Biotechnology, Physiology, Zoology

## Abstract

The development of strategies to overcome the shortage of forage due to persistently low rainfall is becoming a central task for animal nutrition in research and practice. In this study, it was investigated how the treatment of straw with NaOH or feed urea in a practicable procedure for modern farms affects rumen fermentation (gas production and greenhouse gas concentration) as well as the digestibility of feed energy and nutrients. For this purpose, the treatments were tested individually and in different proportions in a total mixed ration (TMR) in ruminal batch cultures in vitro and in a digestibility trial with sheep. In order to explain the observed effects at the molecular level, descriptive data from ^13^C solid state nuclear magnetic resonance (NMR) and Fourier-transform infrared (FTIR) spectroscopy were obtained. NaOH treatment of straw increased crude ash (CA), non-fibrous carbohydrates, digestible energy (DE), and metabolizable energy (ME) concentration, whereas the proportion of neutral detergent fibre (aNDFom) and hemicellulose decreased. In urea treated straw, NH_3_–N and crude protein increased, whereas acid detergent lignin (ADL), DE, and ME decreased. The physically effective fibre (peNDF_8_) concentration increased in TMR containing 18% of NaOH or urea treated straw (*p* < 0.01). The application of straw treatments as pure substrates (not as part of a TMR) increased gas production and decelerated ruminal fermentation (*p* < 0.05). In vitro organic matter digestibility (IVOMD) of the straw (0.31) increased after NaOH (0.51;* p* < 0.05) and urea treatment (0.41; *p* > 0.05). As part of a TMR, straw treatments had no distinct effect on gas production or IVOMD. Concentrations of CH_4_ and CO_2_ were likewise not affected. Apparent total tract digestibility of aNDFom, acid detergent fibre (ADFom), hemicellulose, and cellulose increased in the TMR by approximately 10% points following NaOH treatment (*p* < 0.05). The inclusion of urea treated straw did not affect apparent digestibility. Calculated true digestibility of aNDFom was 0.68, 0.74, and 0.79, of ADFom 0.58, 0.57, and 0.65, and of ADL 0.02, 0.13, and 0.08 in TMR including untreated, NaOH treated, and urea treated straw, respectively. ^13^C NMR and FTIR analyses consistently revealed that the global structure and crystallinity of the carbohydrates (cellulose and hemicellulose) was not altered by treatment and the concentration of lignin was likewise not affected. Depolymerisation of lignin did not occur. However, NMR signals assigned to acetyl groups were significantly altered indicating that straw treatments disrupted linkages between hemicelluloses and lignin. Moreover, the acetates signal was affected. This signal can be assigned to linkages between ferulic acids and hemicelluloses (arabinoxylans). FTIR spectra of straw treatments mainly differed at a wavelength of 1730 cm^−1^ and 1240 cm^−1^. Disappearance of the 1730 cm^−1^ peak suggests removal of hemicelluloses or lignin related compounds by treatment. The disappearance of the lignin peak at 1240 cm^−1^ could be due to conjugated ketone (phenyl-carbonyl) removal or the removal of ferulic and *p*-coumaric acid acetyl groups. Both treatments are supposed to release fermentable cell wall components (hemicelluloses) from lignin-associated bonds and as a result, straw fibre can be better fermented in the rumen. This contributes to energy supply and increased fibre digestibility at least in the TMR that contained NaOH treated straw. The alkaline straw treatments probably induced a release of phenolics such as ferulic acid and *p*-coumaric acid, which can be metabolised in the gut and the liver and metabolites might be excreted with the urine. This could notably contribute to metabolic energy losses.

## Introduction

In the course of an ongoing climate change, regions with significant agricultural production such as Central Europe and Central North America will likely experience a long-term increase of drought frequency and persistence in spring and summer with lasting extraordinary high temperatures and at the same time an increase of events of heavy precipitation^1–4^. This will deeply impair food and feed production due to crop and pasture shortfalls^1^. Such forecasts and actual experiences with forage shortages in the years 2018–2020 in Europe have led to the reactivation of the search for alternative forage resources.

Ruminants are proficient to utilise feeds rich in fibre due to their symbiosis with rumen microbial populations and this is why by-products from the agro-industry and food production, grassland, or woodland can be applied with expected good success^5^. This is also one reason why ruminants will play an essential role in future global land use and food production systems if sustainability goals are to be achieved under the influence of ongoing climate change^6^.

Despite forage shortage, cereal straws remain available to a large extent and throughout the year. In 2020, 60% of barley, 60% of oats, 86% of rye, 34% of wheat, and 94% of triticale produced worldwide came from Europe and this resulted in a total of around 321 million tonnes of straw residue^7,8^. However, straws are poorly digestible, which is due to a concentration of lignin up to 17% of dry matter (DM)^9–13^. Lignin is a major cell wall component that is involved in providing rigidity, internal transport of water and nutrients, and protection^12^. The lignin polymer consists of phenylpropane units, whose precursors are the monolignols *p*-coumaryl alcohol, coniferyl alcohol, and sinapyl alcohol^12^. Those are building a complex amorphous polymer via β-O-4, α-O-4, β-5, β-1, 5–5, 4-O-5, and β-β linkages, which is further forming complexes with other polymers such as cellulose and hemicellulose^12^.

Aside from physical and biological treatments of straw, chemicals can be used to break up the interpolymer bonds in the cell wall in order to release carbohydrates (e.g., hemicelluloses) that rumen microorganisms do readily ferment^14^. The application of alkalis such as NaOH, KOH, Ca(OH)_2_, or urea is associated mainly with improved digestibility^13,15^ and may improve low-quality feeds’ value. Especially NaOH and urea treatments were intensively studied and their effects are well documented^13,15–19^. The degrading effect of alkalis is usually higher the stronger the caustic solution or its degree of dissociation^20^. This makes NaOH more effective and cheaper than KOH or Ca(OH)_2_^20^. The sodagrain method for NaOH application developed by Ørskov et al*.*^21^ is well established in agricultural practice, which is why we decided to build upon this experience. Although urea is less effective than NaOH^20^, it was chosen as it is easy and safe to handle and may improve straws’ feed value by adding nitrogen. In general, application protocols need to be revised against the background of current regulations of the European Union on environmental protection to avoid water and air pollution.

The objectives of this study were to examine effects of NaOH or urea treatment of barley straw on carbohydrate fermentation and in vitro organic matter digestibility (IVOMD) in the rumen and total tract digestibility of energy, crude nutrients, and detergent fibres.

## Methods

### Ethical and policy notes

The animals used in the experiments presented in this report were kept and cared for by the Research Centre for Agricultural and Nutritional Sciences, Martin Luther University Halle-Wittenberg, Wettin/Löbejün, Saxony-Anhalt (Germany). The sheep were used for experimental purposes with the approval of the Saxony-Anhalt Federal Administration Authority (approval no. 42505-3-813 and 2-1524 MLU).

Sampling of plant materials did not involve threatened or wild flora species in terms of the IUCN Policy Statement on Research Involving Species at Risk of Extinction.

All methods described in the following were performed according to the relevant guidelines and regulations as cited.

Use of experimental animals was reported in accordance with the ARRIVE guidelines.

### Barley straw treatments and composition of total mixed rations (TMR)

Untreated winter barley straw served as the control. The same batch of straw was used for the treatments. For NaOH treatment, caustic soda microbeads of technical grade (WHC GmbH, Hilgertshausen, Germany) were dissolved in tap water following the procedure described by Ørskov et al*.*^21^. Then, 60 g NaOH/kg DM of chopped straw (2–5 cm particle size) were applied and mixed for 5 min. Safety measures as recommended by the manufacturer were considered. For the urea treatment, feed grade urea (90%) (SALVANA TIERNAHRUNG GmbH, Kl. O.-Sparrieshoop, Germany) was applied with 40 g/kg DM of the chopped straw and mixed for 5 min. With both treatments, the straw was remoistened to 600 g DM/kg. The treated straw was stored 14 days at ambient temperature in January 2021 (0.8 °C on monthly average) (NaOH treatment) and 25 °C (urea treatment) and then frozen to − 20 °C until analysis. The treatment of the straw, including remoistening, and mixing of the TMR, was carried out in a feed mixer wagon. Untreated straw was also run through the feed mixer and then mixed into the TMR.

A series of experiments was conducted. Initially, an in vitro batch culture experiment was performed to assess treatment effects on gas production as a measure of carbohydrate fermentation in the rumen and IVOMD using untreated barley straw, NaOH, and urea straw treatments. Additionally, a basal TMR consisting of 90 mg barley, 4 mg soybean meal, and 106 mg lucerne chaff proportionately to 200 mg on a DM basis of total substrate was fed in the in vitro fermenters, and six TMR variants in which straw treatments replaced lucerne chaff by 10.6 mg (10%) or 21.2 mg (20%), respectively (Experiment 1). A second batch culture experiment was conducted using practical TMR, which were made of 250 g of the untreated straw or one of the treated straws, 600 g maize silage, 200 g rapeseed meal, 250 g barley, and 200 g water on as fed basis. The straw treatments accounted for approximately 18% in the TMR on DM basis (Experiment 2). These TMR variants were then used to determine total tract digestibility of energy, crude nutrients, and detergent fibres in a digestibility trial with sheep (Experiment 3).

Nutrient composition of the straw treatments used in Experiment 1 and the TMR variants used in Experiment 2 and Experiment 3 is given in Table 1. The barley straw treated with NaOH had 0.6 mmol NH_3_/L extract (50 g straw/400 mL distilled water, 24 h incubation), which is 1.1 g NH_3_–N/kg total nitrogen, and a pH of 10.0. Urea treated straw had 27.6 mmol NH_3_/L, 310 g NH_3_–N/kg total nitrogen, and a pH of 9.1.

**Table 1:** Dry matter and crude nutrient concentrations of untreated barley straw, NaOH treated straw, and urea treated straw (Experiment 1), and in TMR including one of these straw treatments (Experiment 2 and Experiment 3).

Chemical composition (g/kg DM)	Pure substrates (Exp. 1)			TMR (Exp. 2 and Exp. 3)		
	Untreated	NaOH	Urea	Untreated	NaOH	Urea
DM (g/kg)	945	637	690	566	588	592
Crude ash	72	135	72	42	54	44
Crude protein	37	40	67	134	118	141
AEE	5	5	4	22	20	18
Crude fibre	413	410	451	195	215	206
aNDFom	787	661	791	377	395	404
ADFom	503	488	509	227	246	232
ADL	75	73	65	38	40	38
Hemicellulose	287	173	282	150	149	172
Cellulose	428	415	444	189	206	194
NFE	473	410	406	608	594	591
NFC	99	159	66	425	413	393

Practical TMR were composed of 33.5% maize silage, 21.6% rapeseed meal, 26.7% barley grains, and 18.2% of one of the straw treatments on DM basis. The straw treatments and conducted experiments are specified in the text.

ADFom, acid detergent fibre expressed exclusive of residual ash; ADL, acid detergent lignin; AEE, acid ether extract; aNDFom, amylase-treated neutral detergent fibre expressed exclusive of residual ash; DM, dry matter; NFC, non-fibrous carbohydrates; NFE, nitrogen-free extract; TMR, total mixed ration.

### Analysis of particle size distribution and physically effective neutral detergent fibre (peNDF_8_)

For the analysis of particle size distribution of the practical TMR variants, we used a GORR device with a 19- and an 8-mm sieve (GORR GmbH, Eschwege, Germany) similar to the 2-sieve model of the Penn State Particle Separator^22^. We followed the instructions provided by the Penn State University^23^ and performed this analysis twice, first using the original “wet” material with five repetitions for each TMR variant, and secondly, using material dried at 60 °C for 24 h with three, three, and two repetitions for the untreated TMR, NaOH treated TMR, and urea treated TMR, respectively. The physical effectiveness factor (pef) of each of the TMR variants was calculated according to Li et al*.*^24^ as the proportion of DM retained on the 19- and 8-mm sieves of the separator (pef_8_). Then, peNDF_8_ was calculated as pef_8_ × neutral detergent fibre (aNDFom) content^24^.

### In vitro incubations

Ruminal carbohydrate fermentation was analysed using gas production in an in vitro model based on microbial batch cultures, which were sampled from ruminal fluid. For the batch culture experiments, ruminal fluid was obtained from four rumen-cannulated adult German Blackheaded Mutton wethers. The sheep had free access to tap water. Meadow hay was offered ad libitum and each animal received daily 200 g of a 3 mm-pelleted concentrate (IBEKA PANTO Schäferstolz, HL Hamburger Leistungsfutter GmbH, Hamburg, Germany) and 10 g of a mineral feed preparation (basu-kraft® Top-Mineral; BASU Heimtierspezialitäten GmbH, Bad Sulza, Germany). The sheep had the possibility to ingest straw from the bedding. Dry matter and crude nutrient concentrations of hay and the concentrate are given in Table 2. As declared by the manufacturer, the mineral feed was composed of calcium carbonate, sodium chloride, calcium-sodium phosphate, wheat semolina bran, monocalcium phosphate, and sugar beet molasses and contained 190 g calcium, 40 g phosphor, 110 g sodium, and 35 g magnesium/kg on as fed basis. Additives per kg mineral feed were: 3000 mg zinc (2000 mg zinc oxide 3b603 and 1000 mg zinc sulphate, monohydrate 3b605), 820 mg manganese (700 mg manganese(II) oxide 3b502 and 120 mg manganese(II) sulphate, monohydrate 3b503), 250 mg copper (copper(II) sulphate, pentahydrate 3b405), 50 mg iodine (calcium iodate, water-free 3b202), 25 mg selenium (sodium selenite 3b801), and 10 mg cobalt (coated cobalt(II) carbonate granulate 3b304).

**Table 2:** Dry matter and crude nutrient concentrations of meadow hay and the concentrate fed to the sheep used for ruminal fluid collection.

Chemical composition (g/kg DM)	Hay	Concentrate
DM (g/kg)	987	962
Crude ash	83	56
Crude protein	53	49
AEE	3	28
Crude fibre	365	35
aNDFom	654	127
ADFom	400	47
ADL	65	10

ADFom, acid detergent fibre expressed exclusive of residual ash; ADL, acid detergent lignin; AEE, acid ether extract; aNDFom, amylase-treated neutral detergent fibre expressed exclusive of residual ash; DM, dry matter.

In vitro incubations were carried out using the ANKOM RF Gas Production System (ANKOM Technology, Macedon, NY, USA). Experiment 1 was conducted in May 2021 and Experiment 2 in November 2021. Each experiment comprised four consecutive runs (i.e., four biological replicates). In Experiment 1, the substrates (i.e., pure straw treatments or TMR) were incubated in duplicate and four blanks that contained only ruminal fluid and buffer solution were considered in each run; in Experiment 2, substrates were incubated in triplicate and four blanks were considered (i.e., two or three technical replicates per run; four in case of blanks). The batch culture experiments were carried out in accordance with the protocol of the Association of German Agricultural Analytic and Research Institutes (VDLUFA)^25^ using method no. 25.1. Ruminal fluid was collected from two of the four wethers immediately before feeding and about 1 h prior to the start of a run. The animals were randomly selected for taking ruminal fluid. The mixed fluid was filtered through a one-layered cheesecloth and stored in a thermos bottle during transport to the laboratory. The buffer solution was adjusted to prolonged incubation times, which means that 2 g NH_4_HCO_3_ were added and 2 g NaHCO_3_/L were reduced compared to the original recipe^26^. In Experiment 1, the ruminal fluid had a pH of 6.6 ± 0.068, a redox potential of − 264 ± 11.7 mV, and a temperature of 36 ± 0.96 °C when arriving the laboratory. In Experiment 2, the ruminal fluid had a pH of 6.7 ± 0.090, a redox potential of − 264 ± 15.4 mV, and a temperature of 33 ± 2.3 °C. The inoculum was prepared of two parts of the buffer (nutrient solution) and one part of ruminal fluid under stirring and continuous CO_2_ flush. In Experiment 1, the inoculum had a pH of 6.8 ± 0.031 and a redox potential of − 239 ± 8.63 mV. In Experiment 2, the inoculum had a pH of 6.7 ± 0.055 and a redox potential of − 209 ± 12.7 mV. A quantity of 0.2 g of substrate, pulverised using a ball mill (Retsch MM 400, Retsch GmbH, Haan, Germany), was weighed into the fermenters and 30 mL of inoculum was added using an automated pump. We used glass bottles with an actual volume capacity of 136 ± 2.68 mL, which is approximately a 106 mL headspace volume. The fermenters were capped with gas pressure measuring modules and placed into water baths at 80 r/min agitation and a constant temperature of 39 °C. To purge out oxygen, the fermenters were vented with argon through the modules’ Luer port until the inner pressure exceeded 55 kPa, whereupon the entire gas was automatically released. Gas production was measured using the following settings: at least 5 min recording interval, a 10 kPa threshold for the automatic release of accumulated gas in order to prevent supersaturation in the medium, and a valve open time of 150 ms. The cumulative gas pressures were automatically recorded in real-time and afterwards corrected for blank fermentation and converted first to moles of gas produced using the Ideal Gas Law and then to mL of gas using Avogadro's Law.

The fermenters’ headspace gas was sampled at four periods throughout incubation (between 2 and 3 h, 4 and 5 h, 24 and 25 h, and 48 and 49 h) via the modules’ vent valve using an adapter connected to a 2.5 mL gas-proof syringe (SGE Analytical Science, Trajan Scientific and Medical, Ringwood, Australia). In the syringe, vacuum was created, the module was activated manually, and gas flowed into the syringe. At least 2 mL gas was collected per sample and immediately injected into a Shimadzu GC 2010 Plus (Shimadzu Corp., Kyoto, Japan) fitted with a 250 μL upstream gas loop, a Shin Carbon micro-packed column (Restek Corp., Bellefonte, PA, USA; 2 m × 0.53 mm inner diameter, 80/100 mesh size), and a Barrier Discharge Ionization detector (BID-2010 Plus). Helium was the carrier gas. The following settings were applied: 150 °C injection temperature, split mode with a split ratio of 2, 250 kPa pressure, 45.6 mL/min total flow, 14.2 mL/min column flow, 129.8 cm/s linear velocity, 3 mL/min purge flow, oven temperature programme: 35 °C hold for 2.5 min, 20 °C/min to 180 °C, hold for 0.5 min, 280 °C detector temperature, 50 mL/min discharged flow, and a sampling rate of 40 ms. Peak areas of CO_2_, CH_4_, and H_2_ were corrected for corresponding gas peaks that appeared in the blank fermenters. This was only applicable until the second sampling period.

The IVOMD coefficients were calculated on the basis of gas production measured in the batch cultures and crude nutrient analyses as proposed by Menke and Steingass^27^ using equation no. 43f., where IVOMD (%) = 14.88 + 0.8893 gas production (mL/200 mg DM) + 0.0448 crude protein (CP) (g/kg DM) + 0.0651 crude ash (CA) (g/kg DM). Note, gas production was measured using the ANKOM RF Gas Production System and not the Hohenheim Gas Test as proposed by Menke and Steingass^27^. This could have led to small differences in IVOMD estimates.

### In vivo determination of energy and nutrient digestibility

Total tract digestibility of TMR including the straw treatments was determined in a digestibility trial. Twelve adult Pomeranian Coarsewool wethers served as model animals. All animals were clinically healthy and under regular veterinary supervision. The digestibility trial was performed following the guidelines of the Society of Nutrition Physiology (GfE)^28^. The experiment consisted of 14 d adaptation to the diet followed by 6 d of total collection of faeces. Four wethers each received one of the three TMR variants. The animals were randomly assigned to the TMR variants. No inclusion or exclusion criteria were set. Feed bulk samples were collected immediately before the start of the experiment during the weighing of the rations. The sheep additionally received 10 g/day of a mineral feed preparation (basu-kraft® Top-Mineral; BASU Heimtierspezialitäten GmbH, Bad Sulza, Germany) and tap water was offered ad libitum. The actual feeding level of the sheep was 0.80 ± 0.081 times the energy maintenance level (0.43 MJ metabolizable energy, ME/kg body weight^0.75^/day)^28,29^. The feeding level was chosen in deviation from the GfE recommendations in order to ensure that the animals leave as little food as possible, which has proven itself in previous trials. The recommended feed protein content for digestibility determination in sheep is a minimum of 120 g CP/kg diet DM^28^. This was widely achieved in this experiment. The diets were offered in two equal meals per day. During the total collection period, the sheep were individually housed in metabolic cages and were fitted with harnesses to ensure complete faeces collection. The harnesses were emptied each morning prior to the first meal of the day. Daily defecation was quantified and an aliquot of 20% was taken. The quantity of feed residuals was recorded each morning. Feed residuals were minimal 0.13% and maximal 1.9% of the feed quantity that was offered and were considered for digestibility calculations. The faecal samples of each collection day were combined to bulk samples per animal and collection period. Feed and faecal samples were stored dry or were frozen to -20 °C, respectively. The animals were weighed immediately before adaptation, before the collection period, and at the end of the experiment. The initial body weight of the sheep was 64 ± 9.2 kg and remained constant during the experiment (body weight was 61 ± 9.5 kg after adaptation and 62 ± 9.2 kg after the collection period). Digestibility coefficients of DM, organic matter (OM), GE, crude nutrients, and detergent fibres were calculated as (intake—faecal output)/intake over the 6 days of faeces collection for each individual. The determined digestibility coefficients were used to predict digestible energy (DE), and ME and net energy lactation (NEL) concentration of the TMR variants according to GfE^29^, where ME (MJ/kg DM) = 0.0312 digestible acid ether extract (AEE) + 0.0136 digestible crude fibre (CF) + 0.0147 (digestible OM−digestible AEE−digestible CF) + 0.00234 CP. The concentration of NEL was then calculated as follows: NEL (MJ/kg DM) = 0.6 (1 + 0.004 (q − 57)) ME, where q = (ME/gross energy, GE) × 100. Digestibility coefficients of the detergent fibres were additionally determined using linear regression between intake of indigestible detergent fibre and intake of total detergent fibre and calculated as 1 − *b*, where *b* is the slope of the regression line when the function is *y* = *a* + *bx* (*x* is the explanatory variable, *y* is the dependent variable, and *a* is the intercept).

### ^13^C solid state NMR and FTIR analysis

^13^C solid state NMR analysis was performed using a Bruker Avance III HD 300 MHz spectrometer (Bruker Corp., Billerica, MA, USA). A classic (Bruker CP) cross polarization sequence was used at magic angle spinning. The samples were spun in 4-mm zirconia rotors, closed with KeI-F caps, at 12.5 kHz spinning frequency on 4-mm MAS Bruker probe operating. Cross polarization from ^1^H to ^13^C was performed using the Bruker CP method, where the contact time was set at 2 ms, the number of scans was 2048, and D1 was 5 s. Spinal-64 heteronuclear decoupling was applied during the 15-ms acquisition time. The decoupling pulse was optimised on a ^13^C-labelled histidine standard sample and set to 6.4 μs. The NMR data were processed (phase and baseline) by top spin Bruker 4.1.4 software and the spectra are displayed with a line broadening value of 10.

Fourier-transform infrared spectroscopy was acquired using a FTIR ATR Bruker Alpha P spectrometer (Bruker Optics GmbH & Co. KG, Ettlingen, Germany) in the range of 400 to 4000 cm^−1^. The ATR spectra were collected with a 4 cm^−1^ resolution and the results were averaged on 64 scans with manual baseline correction and normalisation.

### Additional chemical analyses

Feed and faeces samples were freeze-dried and ground to pass a 1 mm sieve of a standard laboratory sample mill. Dry matter, CA, CP, AEE, CF, and detergent fibres were analysed using VDLUFA methods no. 3.1, 4.1.1, 5.1.1 B, 6.1.1, 6.5.1, 6.5.2, 6.5.3, and 8.1^25^. Neutral detergent fibre was pre-treated with heat-stable amylase added to the neutral detergent solution. Neutral detergent fibre and acid detergent fibre (ADFom) were expressed exclusive of residual ash. The concentration of GE was determined by bomb calorimetry using a C7000 Oxygen Bomb Calorimeter (IKA Werke, Staufen, Germany). The proportion of nitrogen-free extract (NFE) was calculated as NFE = 1000−CA−CP−AEE−CF. Hemicellulose, cellulose, and non-fibrous carbohydrates (NFC) concentrations were calculated as follows: Hemicellulose = aNDFom−ADFom, cellulose = ADFom−acid detergent lignin (ADL), and NFC = 1000−(CP + aNDFom + AEE + CA). The pH and redox potentials were measured using a Mettler Toledo Seven Excellence unit with InLab® Expert Pro and InLab® Redox electrodes (Mettler Toledo GmbH, Greifensee, Switzerland). The concentration of NH_3_ in feed extracts (50 g straw, 400 mL water, and 24 h incubation time) was determined using the method of Conway and Byrne^30^.

### Statistical analysis

Statistical analysis was performed using SAS 9.4 analytical software (SAS Institute Inc., Cary, NY, USA). The gas production data set was scaled down to a 0.5 h resolution and non-linear regression analysis was performed using the MODEL procedure and Gompertz function^31^. Significant differences in estimated parameters of gas production kinetics among the substrates or treatments were assessed using likelihood ratio 95% confidence intervals. The concentration of peNDF_8_ as well as the concentrations of CO_2_, CH_4_, and H_2_ were compared among the three TMR variants using the Kruskal–Wallis test with the NPAR1WAY procedure. In the gas components data set, outliers were identified using boxplots and removed. Outliers were defined as observations further than 3 times of the interquartile range. The peNDF_8_ contents were compared between “wet” and “dry” sieving, and IVOMD and total tract digestibility coefficients were compared among the straw treatments and/or TMR variants using the Wilcoxon Rank Sum test with NPAR1WAY. Differences with *p* < 0.05 were considered to be significant.

## Results and discussion

### Energy and nutrient concentrations

The nutrient concentrations of the pure straw treatments and the TMR variants are given in Table 1 and calculated energy concentrations are given in Table 3. Energy and nutrient concentrations are provided as descriptive information and were not subjected to statistical evaluation. However, there were some noticeable changes as a result of straw treatments and trends, which are described in the following. In NaOH treated straw, the concentration of CA increased by 6% points, concentrations of aNDFom and hemicellulose decreased by 13 and 11% points, respectively, the concentration of NFC increased by 6% points, and as a result, DE and ME concentration of the TMR increased by 0.2 MJ/kg DM. An increased CA concentration (here from 72 to 135 g/kg DM) usually reduces OM concentration and the energy density of the straw. The addition of 60 g NaOH/kg straw DM corresponds to a sodium concentration of about 30 g/kg DM^32,33^, which is a significant increase compared to untreated straw (0.2 g/kg DM)^33^. After aerobic storage of the straw, a part of sodium is present as NaHCO_3_, which remains as part of the inorganic residue during incineration and is recorded as CA. Rogers et al*.*^34^ and Masters et al*.*^35^ reported feed intake depression and declined digestibility as a result of high sodium levels in the rumen and increased osmolality of the ruminal fluid. As part of the practical TMR formulations used in this study (18.2% inclusion on DM basis), NaOH treated straw did not induce a distinct CA increase (just a plus of 1.2% points) and thus, it had no detrimental effect on DE or ME concentration of the diet or on digestibility. In urea treated straw, the CP concentration was higher than in untreated straw (3% points; total nitrogen contained 31% NH_3_–N). This TMR with 18.2% urea treated straw with 40 g urea/kg straw (i.e., 18.4 g nitrogen/kg straw with 46% nitrogen in the urea) and 141 g CP/kg DM (i.e., 22.6 g nitrogen), contained 14.8% nitrogen from urea, measured against the total nitrogen in the ration. Thus, this TMR was in a range where the supplemental nitrogen source may have had a positive effect on microbial growth in the rumen and microbial protein and fatty acid synthesis, as well as on the digestibility of DM, OM, detergent fibres, and CP^36–38^. However, urea concentrations significantly higher than this would burden the liver metabolism, through which excess NH_3_ has to be detoxified, and lead to increased nitrogen excretion via the urine^39^. Urea treatment of the straw did also decrease ADL concentration of the TMR by 10% points and the DE and ME concentration by 0.5 and 0.1 MJ/kg DM, respectively. The results are consistent with previous reports^40,41^.

**Table 3:** Energy concentration of TMR composed of 33.5% maize silage, 21.6% rapeseed meal, 26.7% barley grains, and 18.2% of untreated straw, NaOH treated straw, or urea treated straw on DM basis.

Energy level (MJ/kg DM)	Untreated	NaOH	Urea
GE	18.5	18.1	18.1
DE	13.9	14.1	13.4
ME	11.3	11.5	11.2
NEL	6.9	7.1	6.9

The straw treatments are specified in the text. GE was determined by bomb calorimetry, DE was calculated on the basis of GE digestibility determined in the digestibility trial, ME was calculated on the basis of crude nutrient digestibility determined in the digestibility trial, and NEL was calculated on the basis of GE and ME according to GfE^29^.

DE, digestible energy; DM, dry matter; GE, gross energy; ME, metabolizable energy; NEL, net energy lactation; TMR, total mixed ration.

### Particle size distribution and peNDF_8_

**Figure 1:**
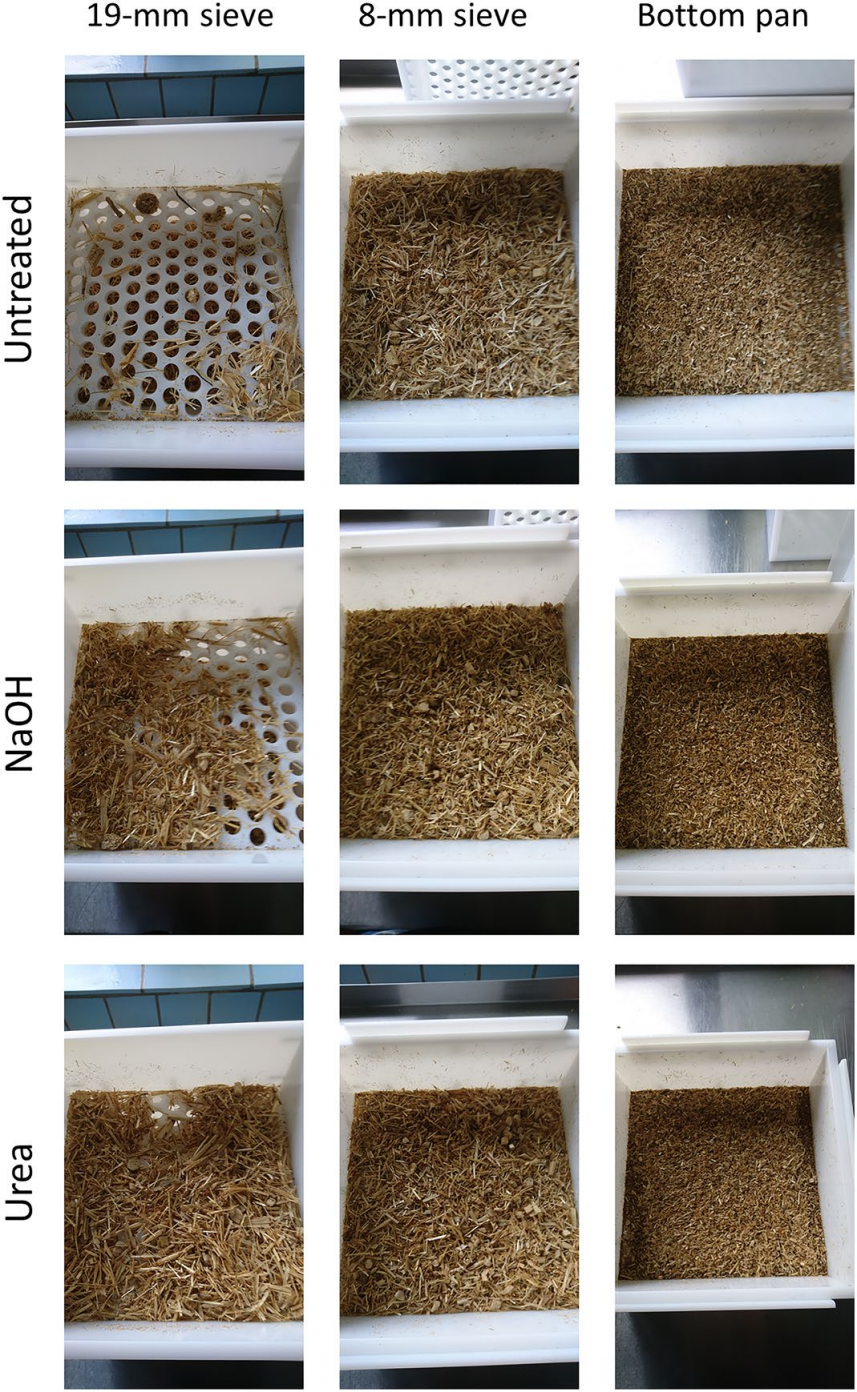
Illustration of particle size distribution of as fed “wet” TMR composed of 33.5% maize silage, 21.6% rapeseed meal, 26.7% barley grains, and 18.2% of untreated, NaOH treated, or urea treated barley straw on dry matter basis using the method described by Lammers et al.^22^ and Heinrichs and Jones^23^. TMR, total mixed ration.

Particle size distribution of the as fed TMR variants is illustrated in Fig. 1. In the TMR containing untreated straw, 2 ± 0.5% of particles were retained on the upper sieve of the separator (19 mm), 31 ± 1.4% on the middle sieve (8 mm), and 67 ± 1.8% were smaller than 8 mm and found on the bottom pan, in the TMR containing NaOH treated straw, 8 ± 5% of particles were larger than 19 mm, 31 ± 2.4% were between 19 and 8 mm, and 61 ± 2.9% were smaller than 8 mm, and in the TMR containing urea treated straw, 17 ± 5.0% were retained on the upper sieve, 22 ± 3.5% on the middle sieve, and 61 ± 1.9% of particles laid on the bottom pan. Then, pef_8_ and peNDF_8_ were 0.33 ± 0.018 and 124 ± 6.66 g/kg DM in the TMR with untreated straw, 0.39 ± 0.029 and 153 ± 11.4 g/kg DM in the TMR with NaOH treated straw, and 0.39 ± 0.019 and 156 ± 7.47 g/kg DM in the TMR with urea treated straw, respectively. The peNDF_8_ of TMR significantly increased in the variants containing NaOH or urea treated straw (*p* < 0.01). The 19- and 8-mm sieves retain particles from the diet which are buoyant in the rumen, form the forage mat, substantially support rumination and pH regulation (prevention of acidosis), and promote fibre digestion^23,42^. On that basis, the diet’s peNDF_8_ concentration is a key factor maintaining physiological ruminal function^42^. In dairy cows, peNDF_8_ should have a proportion of 15–18% in the diet on a DM basis, when starch is between 20 and 25% and NFC are between 35 and 40%^43^. The higher content of peNDF_8_ in the TMR with treated straws was mainly due to an accumulation of large particles retained by the 19-mm sieve. To clear up if this could be a result of remoistening during processing in the feed mixer, we performed the analysis a second time using dried materials (dried at 60 °C for 24 h; 967 ± 29.0 g DM, 995 ± 0.509 g DM, and 978 ± 29.9 g DM/kg in untreated, NaOH treated, and urea treated straw TMR, respectively). In the dried TMR, peNDF_8_ was 95 ± 3.9 g/kg (untreated), 130 ± 25.9 g/kg (NaOH treated), and 113 ± 14.6 g/kg (urea treated). This was consistently lower than with “wet” sieving (*p* < 0.05 for the untreated TMR, *p* > 0.05 for the treatments). Among the dried TMR variants, peNDF_8_ contents did not significantly differ, but showed a clear trend to an increase after treatment. There was no indication of a relevant remoistening effect, but alkaline treatment in the feed mixer could have led to swelling of fibre structures (e.g., cellulose)^44^ and particle agglomeration, which was not reversed after drying.

### In vitro carbohydrate fermentation—gas production and composition

**Figure 2:**
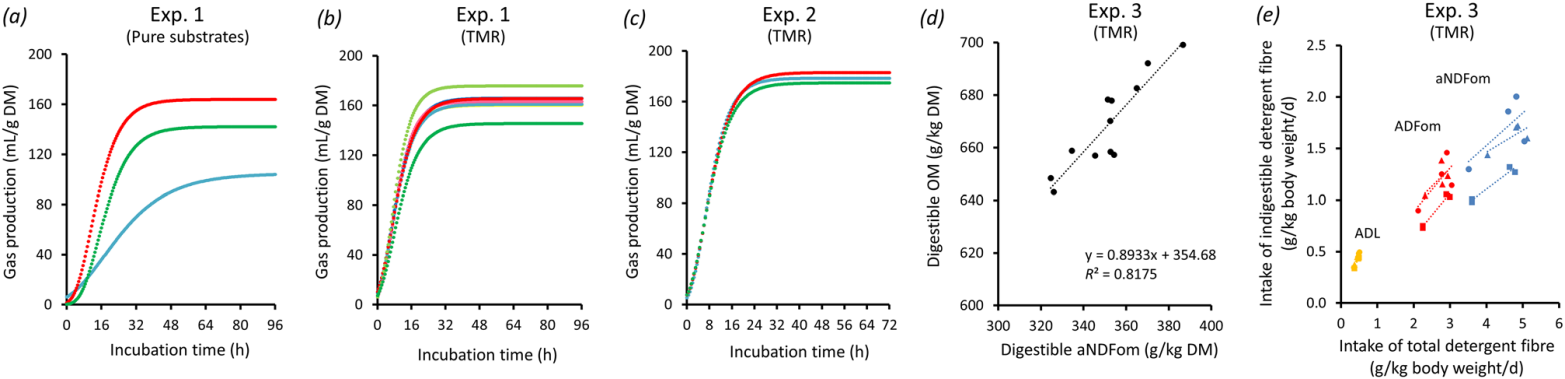
Gas production profiles from incubation of pure straw treatments (**a**), a model TMR composed of 45% barley grains, 2% soybean meal, and 53% lucerne chaff and model TMR including the straw treatments with 10 or 20% of the proportion of lucerne chaff (Experiment 1) (**b**), and practical TMR composed of 33.5% maize silage, 21.6% rapeseed meal, 26.7% barley grains, and 18.2% of one of the straw treatments on DM basis in the in vitro batch culture system (Experiment 2) (**c**). Linear relationship between digestible OM and digestible aNDFom concentration of the practical TMR (Experiment 3) (**d**). Linear regression between intake, by sheep, of indigestible detergent fibre and intake of total detergent fibre from the practical TMR (Experiment 3) (**e**). Legend: (**a**, **c**)  untreated straw,  NaOH treated straw,  urea treated straw; (**b**)  basal TMR,  untreated straw (10%),  untreated straw (20%),  NaOH treated straw (10%),  NaOH treated straw (20%),  urea treated straw (10%),  urea treated straw (20%). The straw treatments and conducted experiments are specified in the text. ADFom, acid detergent fibre expressed exclusive of residual ash, ADL, acid detergent lignin; aNDFom, amylase-treated neutral detergent fibre expressed exclusive of residual ash; DM, dry matter; OM, organic matter; TMR, total mixed ration.

Estimated parameters of gas production kinetics and goodness of fit indicators are summarized in Table 4 and the curves are illustrated in Fig. 2a–c. In Experiment 1, the asymptote of gas production from fermentation of untreated barley straw was at 105 mL/g DM and it lasted about 17 h to achieve one-third and 33 h to achieve two-third of the maximal gas production. This is within the range of gas production profiles obtained from incubations of barley straw during a ring test^45^. Gas production was significantly increased in NaOH and urea treatments (*p* < 0.05) and fermentation was decelerated (*p* < 0.05). This was more distinct in NaOH than in urea treated straw (*p* < 0.05). Previous studies have shown that alkaline treatments of straw are able to break down hemicellulose-lignin and lignocellulose bonds, hydrolyse uronic and acetic acid esters, and disrupt cellulose crystallinity, leading to improved degradation of hemicelluloses and cellulose^13,14,46,47^ and this is reflected by increased gas production as a direct measure of microbial activity^48^. In the TMR that contained untreated straw by 10% of lucerne chaff, gas production profiles did not differ from that of the basal TMR. Addition of straw by 20% of lucerne chaff increased the gas production asymptote (*p* < 0.05). In the TMR that contained NaOH treated straw by 10% of lucerne chaff, gas production increased compared to the TMR with untreated straw at the 10% level (*p* < 0.05). With a 20% replacement, however, gas production did not differ. The duration of TMR fermentation was barely affected with the NaOH straw treatment. Replacement of lucerne chaff by 10% urea treated straw distinctly increased gas production and hastened fermentation (*p* < 0.05). A further 10% point plus of urea treated straw, however, decreased gas production and deferred fermentation of the TMR (*p* < 0.05). This could have been the result of an asynchronous supply of nitrogen and energy at the beginning of the fermentation period due to excess nitrogen in the system. However, the microorganisms in the batch culture actually recover quickly—within approximately 12 h^49^. In vitro incubation of practical TMR revealed that inclusion of NaOH treated straw increased the gas production asymptote compared to TMR with an untreated straw component (*p* < 0.05; Table 4, Fig. 2c). Gas production decreased by inclusion of urea treated straw (*p* < 0.05; Table 4, Fig. 2c). With both treatments, gas production was decelerated (*p* < 0.05; Table 4). However, on the TMR level, these changes are not relevant from a practical point of view.

**Table 4:** Estimated model parameters for gas production kinetics of barley straw treatments, a model TMR composed of 45% barley grains, 2% soybean meal, and 53% lucerne chaff, the model TMR including the straw treatments with 10 or 20% of the proportion of lucerne chaff (Experiment 1), and practical TMR composed of 33.5% maize silage, 21.6% rapeseed meal, 26.7% barley grains, and 18.2% of one of the straw treatments on DM basis (Experiment 2).

Treatment	*a*	*b*	*c*	*b* + *c*	RMSE	*R*^2^
**Pure substrates (Exp. 1)**						
Untreated	105 (103.9 105.4)	17.1 (16.7 17.4)	16.3 (15.8 16.9)	33.4	2.65	0.993
NaOH	**164 (163.3 164.4)**	*11.2 (11.0 11.3)*	*7.3 (7.1 7.5)*	18.5	3.04	0.996
Urea	**142 (141.6 142.5)**	*14.6 (14.5 14.7)*	*8.0 (7.8 8.2)*	22.6	2.29	0.998
**TMR (Exp. 1)**						
Basal TMR	160 (159.7 161.0)	6.6 (6.4 6.7)	5.9 (5.7 6.1)	12.5	3.84	0.990
Untreated 10%	161 (160.2 161.6)	6.5 (6.3 6.7)	6.2 (5.9 6.4)	12.7	4.24	0.987
Untreated 20%	166 (165.2 166.4)	6.5 (6.3 6.6)	6.2 (6.0 6.4)	12.7	3.58	0.991
NaOH 10%	**163 (162.5 163.8)**	**6.7 (6.5 6.8)**	*5.6 (5.4 5.8)*	12.3	3.59	0.991
NaOH 20%	166 (164.8 166.1)	6.6 (6.4 6.7)	6.4 (6.2 6.7)	13.0	3.79	0.990
Urea 10%	**176 (175.0 176.4)**	6.4 (6.2 6.5)	*5.2 (5.1 5.5)*	11.6	4.26	0.989
Urea 20%	*145 (144.9 146.0)*	**7.4 (7.3 7.6)**	**6.8 (6.6 7.0)**	14.2	3.16	0.992
**TMR (Exp. 2)**						
Untreated	179 (178 179)	6.2 (6.1 6.3)	4.9 (4.8 5.1)	11.1	2.58	0.997
NaOH	**183 (182 184)**	6.4 (6.3 6.5)	**5.5 (5.3 5.7)**	11.9	3.26	0.995
Urea	*175 (174 175)*	6.1 (6.0 6.3)	**5.4 (5.3 5.6)**	11.5	2.89	0.996

Gas production curves were modelled using Gompertz non-linear regression function according to Dhanoa et al*.*^31^. Likelihood ratio 95% confidence intervals are given in brackets. Values that are significantly higher than the untreated counterpart are highlighted in bold, values that are significantly lower are highlighted in italics. Barley straw treatments were untreated straw, NaOH treated straw, and urea treated straw and are specified in the text. The conducted experiments are specified in the text. *a* = asymptotic maximal gas production (mL/g DM), *b* = time (h) until which one-third of *a* is produced, *c* = time (h) between *b* and the time (h) *b* + *c*, until which approximately 70% of *a* is produced, RMSE, root mean square error; TMR, total mixed ration.

Concentrations of CH_4_ and CO_2_ increased with incubation time. In the TMR including untreated straw, 0.6 ± 0.02% CH_4_ and 0.9 ± 0.07% CO_2_ were found in the headspace gas after around 2 h, 1.1 ± 0.67% CH_4_ and 1.7 ± 1.2% CO_2_ were found after 4 h, 3.0 ± 2.1% CH_4_ and 12.5 ± 8.27% CO_2_ were found after 24 h, and 5.3 ± 0.52% CH_4_ and 16.5 ± 0.347% CO_2_ were found after 48 h of incubation. The concentration of gaseous H_2_ was consistently lower than 0.01%. We have no information about the concomitant concentration of H_2_ dissolved in the inoculum, but it could have been many times higher, because in in vitro systems, gaseous and dissolved H_2_ are not necessarily in equilibrium^50^. No differences in gas composition were detected between the incubated TMR including NaOH or urea treated straw, or in comparison to the untreated variant.

### Energy and nutrient digestibility

The IVOMD coefficients provided in Table 5 indicate the theoretical digestibility after feed passage through the rumen and are calculated on the basis of gas production and crude nutrient analyses^27^. In the pure straws (Experiment 1), NaOH and urea treatments increased IVOMD by 20% points (*p* < 0.05) and 10% points (*p* > 0.05), respectively. As part of the TMR (Experiment 2), however, treated straw did not affect IVOMD.

**Table 5:** In vitro organic matter digestibility coefficients (ruminal digestibility) determined in untreated barley straw, NaOH treated straw, and urea treated straw (Experiment 1), and in TMR including one of these straw treatments (Experiment 2) according to Menke and Steingass^27^ (equation no 43f.) on the basis of gas production and crude nutrient analyses.

Pure substrates (Exp. 1)			TMR (Exp. 2)		
Untreated	NaOH	Urea	Untreated	NaOH	Urea
0.31 ± 0.054^b^	0.51 ± 0.043^a^	0.41 ± 0.016^ab^	0.54 ± 0.025	0.54 ± 0.034	0.53 ± 0.030

Practical TMR were composed of 33.5% maize silage, 21.6% rapeseed meal, 26.7% barley grains, and 18.2% of one of the straw treatments on DM basis. The straw treatments and conducted experiments are specified in the text.

DM, dry matter; TMR, total mixed ration.

^ab^Superscripts indicate differences among the treatments (*p* < 0.05).

Total tract DM, OM, energy, crude nutrient, and detergent fibre digestibility coefficients are shown in Table 6 and were widely within the range reported by previous studies using different kinds of straws and different levels of inclusion into a TMR^51–54^. Total tract digestibility was generally highest in the TMR containing NaOH treated straw. The digestibility of aNDFom, ADFom, and hemicellulose of the TMR containing NaOH treated straw increased by approximately 10% points compared to the untreated straw TMR variant (*p* < 0.05). A 9% points plus in digestibility was found for cellulose (*p* = 0.057). The increase in OM digestibility was less pronounced than the increase in fibre digestibility, although there was no reduction in digestibility of any other crude nutrient. Nevertheless, a clear linear relationship between the proportion of digestible OM and the proportion of digestible aNDFom (as an example digestible fibre fraction) was confirmed (Fig. 2d). The release and degradation of hemicelluloses and cellulose following NaOH treatment led to improved energy, OM, and fibre digestibility also in previous studies^16,17,20,47,54,55^. The inclusion of urea treated straw did not notably affect digestibility of energy, ash, OM, nutrients, or fibres in the TMR (Table 6) and this confirms reports that urea is not as much effective as NaOH can be^32,54^. The level of inclusion of treated straw into the diet is crucial for successful application, because more than 30% of alkaline treated straw in a TMR on DM basis may lead to intake depression and body weight decline^56^.

**Table 6:** Digestibility coefficients of dry matter, organic matter, crude nutrients, detergent fibres, and gross energy of total mixed rations including 18% untreated barley straw, NaOH treated, or urea treated barley straw.

Item	Untreated	NaOH	Urea
Dry matter	0.74 ± 0.034^ab^	0.78 ± 0.009^a^	0.74 ± 0.010^b^
Organic matter	0.77 ± 0.031^ab^	0.80 ± 0.008^a^	0.77 ± 0.009^b^
Crude protein	0.71 ± 0.039	0.70 ± 0.022	0.69 ± 0.068
Acid ether extract	0.67 ± 0.150	0.75 ± 0.091	0.67 ± 0.051
Crude fibre	0.62 ± 0.054^b^	0.74 ± 0.012^a^	0.66 ± 0.021^b^
aNDFom	0.63 ± 0.047^b^	0.72 ± 0.009^a^	0.66 ± 0.022^b^
ADFom	0.56 ± 0.053^b^	0.66 ± 0.017^a^	0.55 ± 0.040^b^
Hemicellulose	0.72 ± 0.049^b^	0.83 ± 0.028^a^	0.79 ± 0.056^ab^
Cellulose	0.68 ± 0.055^ab^	0.77 ± 0.022^a^	0.69 ± 0.012^b^
NFE	0.83 ± 0.023^ab^	0.85 ± 0.009^a^	0.82 ± 0.013^b^
Gross energy	0.75 ± 0.033^ab^	0.78 ± 0.012^a^	0.74 ± 0.011^b^

Coefficients are given as mean ± standard deviation. Treatments are specified in the text.

ADFom, acid detergent fibre expressed exclusive of residual ash; aNDFom, amylase-treated neutral detergent fibre expressed exclusive of residual ash; NFE, nitrogen-free extract.

^ab^Superscripts indicate differences among the treatments (*p* < 0.05).

Linear regression analysis was performed between intake of indigestible detergent fibre and intake of total detergent fibre (Fig. 2e). Then, digestibility, calculated as 1 − *b*, of aNDFom was 0.68, 0.74, and 0.79, digestibility of ADFom was 0.58, 0.57, and 0.65, and digestibility of ADL was 0.02, 0.13, and 0.08 in TMR composed with untreated straw, NaOH treated straw, and urea treated straw, respectively. The results of this regression analysis can be interpreted as the true digestibility coefficients of the fibre fractions^57^. They may differ from the apparent digestibility coefficients we obtained from the digestibility trial, however, notable endogenous losses of fibrous carbohydrates are not to expect. The number of available data points, their distribution, and range are decisive for the reliability of the regression approach and must be critically examined in the present case.

### ^13^C NMR and FTIR spectra

To clear up the observed effects of NaOH or urea treatment of straws on ruminal carbohydrate fermentation and on digestibility of fibres, samples of the straw treatments were subjected to ^13^C solid state NMR and FTIR spectroscopy. ^13^C NMR and FTIR analyses were performed using representative bulk samples of the TMR variants. The results of these analyses clearly indicate how the treatment of straw with NaOH or urea works at the molecular level, but are descriptive and do not yet allow statistically reliable statements.

**Figure 3:**
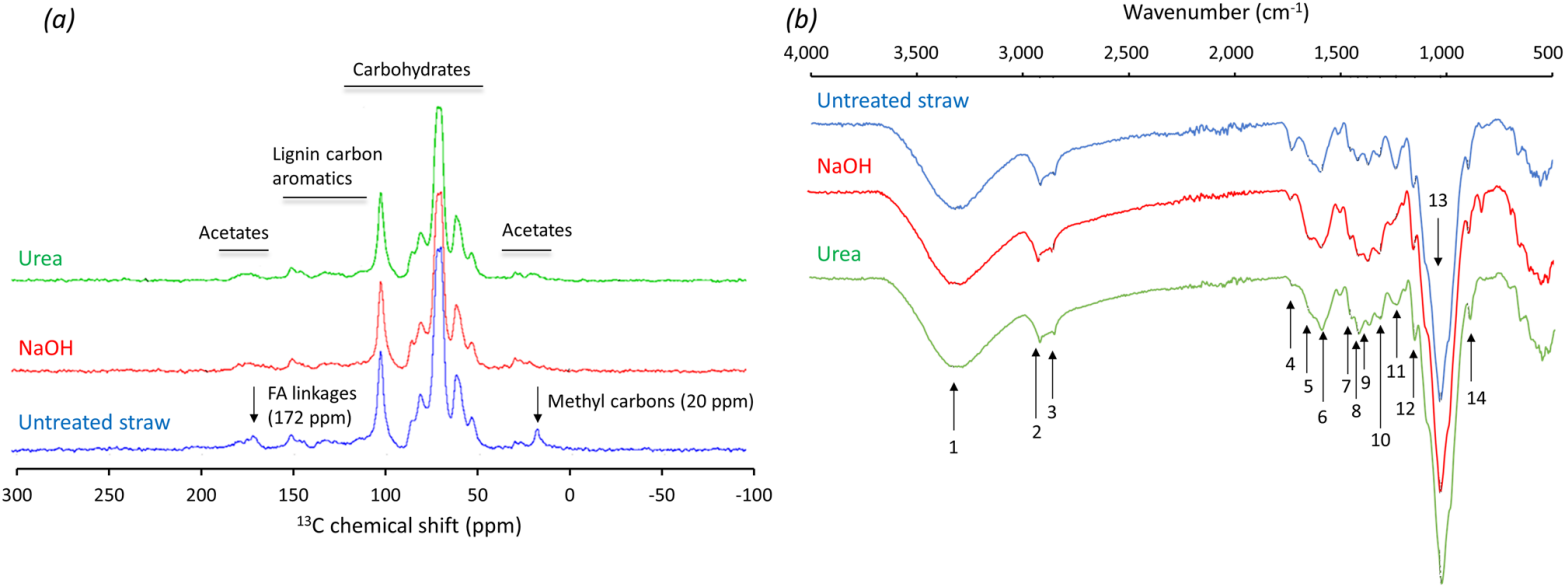
^13^C nuclear magnetic resonance (NMR) (**a**) and Fourier-transform infrared (FTIR) spectra (**b**) of untreated, NaOH treated, and urea treated barley straw. Straw treatments are specified in the text. The band assignments for FTIR spectra (peaks 1–14) are given in Table 7. FA, ferulic acid.

Figure 3a shows the ^13^C NMR spectra for untreated barley straw in comparison to the treatments. The global structure of the carbohydrates (cellulose and hemicellulose) was not altered by treatment. There was no significant variation of the signals, which shows that no hydrolysis of these carbohydrates occurred. There was further no signal broadening observed for the carbohydrates showing that no variation in the ordered structure (crystallinity) had been induced. Variation in lignin structure was not clearly observed. This shows that no important depolymerisation of lignin occurred, which is especially related to ether β-O-4 linkages attributed to syringyl units (at 153 ppm). Signals of OCH_3_ regions at 56.2 ppm and aromatic components at 153–106 ppm did not show any significant variation indicating that also lignin content was not affected by treatment. The signal of methyl carbons at 20 ppm is attributed to methyl carbons in the acetyl groups of hemicelluloses and this signal was significantly modified by the treatments. Hydrolysis of the acetyl groups during treatment leads to the removal of acetic acid. This confirms Ternrud et al*.*^47^, who supposed removal of acetyl groups as one major effect of alkaline straw treatment. The acetates signal at 172 ppm was significantly affected as well (Fig. 3a). This signal could be assigned to linkages between ferulic acids and hemicelluloses (arabinoxylans) described as 8–5’ linkages between ferulic acid and guaiacyl^58^. Ferulates cross-link arabinoxylans and are an important factor in lignification of cell walls^59^. The removal of methyl and acetate groups by NaOH treatment is generally consistent with previous results^60^.

The FTIR spectra of the TMR including untreated barley straw, NaOH treated, or urea treated straw, respectively, are shown in Fig. 3b. The peak assignments are presented in Table 7. The frequencies corresponding to functional groups of lignin, hemicelluloses, and cellulose have been assigned. The position and shape of the OH group did not change significantly, which means that intermolecular interactions between functional group compounds remained intact^61^. The peaks at 2920 and 2840 cm^−1^ correspond to C–H stretching of methyl and methylene^62^. The peak at 1730 cm^−1^ was assigned to C=O stretching from unconjugated ketone, carbonyl, and ester groups of carbohydrate origin^63^. The peak at 1595 cm^−1^ is characteristic for aromatic rings and is the result of vibrations of the aromatic skeleton^64^. Wavenumbers between 800 and 1440 cm^−1^ were attributed to bonds present in multiple plant cell wall polymers^65^. In this region, absorption bands result from multiple bond type vibrations^62,65,66^, specified as follows: 1440 cm^−1^: O–H plane bending, 1420 cm^−1^: C–H plane deformation in lignin, 1370 cm^−1^: —C–H bending in wood, 1320 cm^−1^: C–O syringyl ring and CH_2_ wagging, 1240 cm^−1^: conjugated ketone (phenyl-carbonyl) in lignin, 1160 cm^−1^: C–O–C asymmetrical stretching in cellulose and hemicelluloses, 1030 cm^−1^: C–O, C=C, and C–C–O stretching in wood, and 890 cm^−1^: glycosidic linkage. The main differences between the three FTIR spectra were dedicated to peaks number 4 (1730 cm^−1^) and number 11 (1240 cm^−1^). Peak number 4 disappeared after treatment of straw suggesting that some hemicelluloses or lignin related compounds have been removed^67^. The disappearance of the lignin peak at 1240 cm^−1^ could be due to conjugated ketone (phenyl-carbonyl) removal (lignin that is wrapping the surface of cellulose fibres). Furthermore, this peak disappearance could be due to a ferulic and *p*-coumaric acid acetyl group removal^67^. This observation agreed with the results obtained from ^13^C solid state NMR analysis.

**Table 7:** Band assignments for infrared spectra. Wavenumber is given as cm^-1^.

Peak number	Wavenumber	Assignment/functional group	Component	References
1	3400	O–H stretching	Lignin	Xu et al.^66^
2	2920	C–H stretching	Wood	Xu et al.^66^
3	2840	C–H stretching	Wood	Xu et al.^66^
4	1730	Unconjugated ketone, carbonyl, and ester groups of carbohydrate origin	Lignin	Faix^63^
5	1640	Adsorbed water scissoring, C=C stretching	Lignin	Bahng et al.^62^
6	1595	Aromatic C=C stretch, C=O stretching	Lignin	Bahng et al.^62^, Faix^63^, Nada et al.^64^
7	1440	O–H plane bending	Wood	Sills^65^
8	1420	C–H plane deformation	Lignin	Xu et al.^66^
9	1370	C–H bending	Wood	Xu et al.^66^
10	1320	C–O syringyl ring and CH_2_ wagging	Lignin, wood	Xu et al.^66^
11	1240	Conjugated ketone (phenyl-carbonyl)	Lignin	Bahng et al.^62^
12	1160	C–O–C asymmetrical stretching	Cellulose, hemicelluloses	Xu et al.^66^
13	1030	C–O, C=C, C–C–O stretching	Wood	Xu et al.^66^
14	890	Glycosidic linkage	Cellulose, hemicelluloses	Xu et al.^66^, Barman et al.^75^

It is very likely that ferulic acid, *p*-coumaric acid, and possibly other hydroxycinnamic acids are then present in a free, dissolved form^68,69^. In the forestomach system of ruminants, hydroxycinnamic acids can be metabolised if they are still esterified or etherified (bound) in plant cell wall fragments^70–72^. However, if there is a larger proportion of dissolved phenolic conjugates after straw treatment, these are probably metabolised more quickly and with priority by rumen microorganisms. Ingested phenolics initially undergo rapid hydrogenation and dehydroxylation, resulting in 3-phenylpropionic acid^70^. This is absorbed and metabolised to benzoic acid by β-oxidation in the liver, or is metabolised via cinnamic acid to cinnamoylglycine and renally excreted in this form^70,73^. Benzoic acid is largely—to approximately 75–95%—excreted renally as hippuric acid^70^. Hippuric acid contributes significantly to the renal energy losses in ruminants (0.30 MJ/g renal nitrogen; for comparison: 0.023 MJ/g renal nitrogen from urea), which are 0.33–0.84 MJ/L and thus high per se^74^. A forced release of phenolic compounds through pre-digestion of the straw component in the diet could then have a detrimental effect on the energy balance of the animals.

## Conclusions

Since cereal straws are available in sufficient quantities even when forage is scarce, they offer an important additional source of fibre and, if treated correctly, potentially also an additional source of energy for the nutrition of ruminants. Treatment of straw with NaOH or feed-grade urea can be practicably and safely carried out on farm. Both treatment methods probably led to a release of fermentable cell wall components (hemicelluloses, cellulose, and phenolic acids) from lignin-associated bonds. As a result, straw fibre seems to be fermented more effectively in the rumen and may contribute to the energy supply of the animal. However, the amount of straw in general and the amount of treated straw used in a TMR must be calculated in such a way that there is no decline in feed intake or digestibility. This has to be considered especially with NaOH treated straw, since sodium from added NaOH accumulates in the form of NaHCO_3_ and increases the CA content significantly. An increased CA content then reduces energy density. Digestibility of fibre fractions increased by 10% points in a TMR containing NaOH treated straw. The concentrations of DE, ME, and NEL increased by 0.2 MJ/kg DM. Urea treatment did not affect digestibility or NEL concentration of the TMR, but decreased the concentration of DE and ME by 0.5 and 0.1 MJ/kg DM, respectively. Another fact that needs to be considered is that metabolites from free phenolics such as ferulic acid and *p*-coumaric acid released by treatment could possibly increase metabolic energy losses when excreted with the urine.

## Data Availability

The datasets generated during and/or analysed during the current study are available from the corresponding author on reasonable request.
